# States of quinolinic acid excess in urine: A systematic review of human studies

**DOI:** 10.3389/fnut.2022.1070435

**Published:** 2022-12-16

**Authors:** Marie Christelle Saade, Amanda J. Clark, Samir M. Parikh

**Affiliations:** ^1^Division of Nephrology, Department of Medicine, University of Texas Southwestern, Dallas, TX, United States; ^2^Division of Pediatric Nephrology, Department of Pediatrics, University of Texas Southwestern, Dallas, TX, United States; ^3^Department of Pharmacology, University of Texas Southwestern, Dallas, TX, United States

**Keywords:** quinolinic acid (QA), tryptophan, urine, NAD+, inflammation, NAD+ biosynthesis, metabolism, quinolinate phosphoribosyl transferase (QPRT)

## Abstract

**Introduction:**

Quinolinic acid is an intermediate compound derived from the metabolism of dietary tryptophan. Its accumulation has been reported in patients suffering a broad spectrum of diseases and conditions. In this manuscript, we present the results of a systematic review of research studies assessing urinary quinolinic acid in health and disease.

**Methods:**

We performed a literature review using PubMed, Cochrane, and Scopus databases of all studies reporting data on urinary quinolinic acid in human subjects from December 1949 to January 2022.

**Results:**

Fifty-seven articles met the inclusion criteria. In most of the reported studies, compared to the control group, quinolinic acid was shown to be at increased concentration in urine of patients suffering from different diseases and conditions. This metabolite was also demonstrated to correlate with the severity of certain diseases including juvenile idiopathic inflammatory myopathies, graft vs. host disease, autism spectrum disorder, and prostate cancer. In critically ill patients, elevated quinolinic acid in urine predicted a spectrum of adverse outcomes including hospital mortality.

**Conclusion:**

Quinolinic acid has been implicated in the pathophysiology of multiple conditions. Its urinary accumulation appears to be a feature of acute physiological stress and several chronic diseases. The exact significance of these findings is still under investigation, and further studies are needed to reveal the subsequent implications of this accumulation.

## Introduction

**FIGURE 1:**
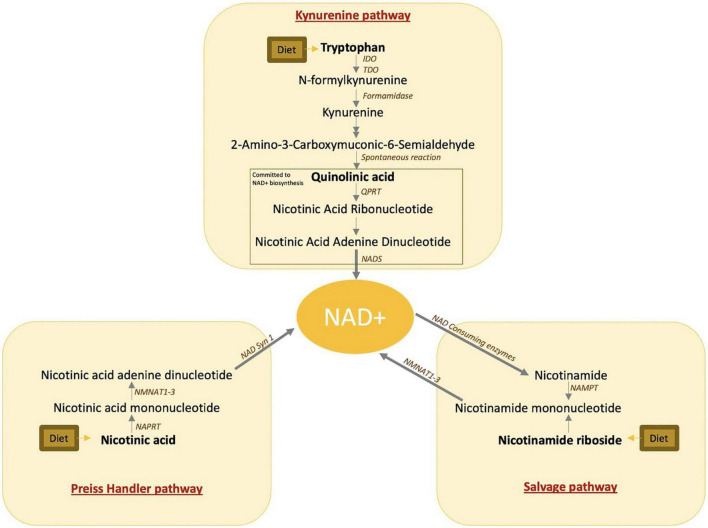
NAD+ biosynthetic pathways. IDO, indoleamine-pyrrole 2,3-dioxygenase; TDO, tryptophan 2,3-dioxygenase; QPRT, quinolinate phosphoribosyltransferase; NAD, nicotinamide adenine dinucleotide; NADS, nicotinamide adenine dinucleotide synthetase 1; NMNAT, nicotinamide mononucleotide adenylyl transferase; NAPRT, nicotinate phosphoribosyltransferase; NAMPT, nicotinamide phosphoribosyltransferase.

**FIGURE 2:**
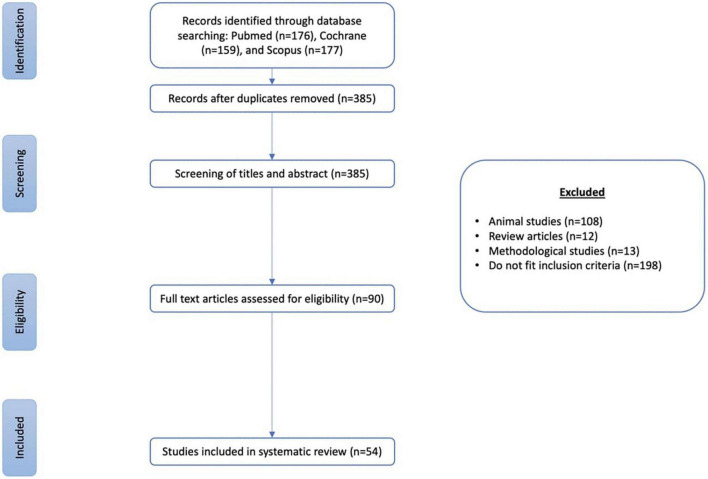
Studies selection flow diagram.

Quinolinic acid, or pyridine-2,3-dicarboxylic acid, was originally described in 1949 by Henderson et al. as a normal component of urine and a possible substrate of tryptophan (1). More recently, quinolinic acid has been recognized as a metabolite of the kynurenine pathway, also known as the *de novo* nicotinamide adenine dinucleotide (NAD+) synthesis pathway. This pathway catabolizes dietary tryptophan into NAD+ and is one of the three independent pathways for NAD+ biosynthesis (Figures 1, 2). In times of stress and inflammation, quinolinic acid accumulates (3, 4). This accumulation was hypothesized to result from two phenomena. First, pro-inflammatory cytokines activate the first and rate-limiting enzyme of the kynurenine pathway, indoleamine 2,3-dioxygenase (IDO), inducing the accumulation of downstream metabolites (5). Second, the quinolinate phosphoribosyl transferase (QPRT) enzyme which catalyzes quinolinic acid and commits the pathway to NAD+ biosynthesis declines or saturates with inflammation (6, 7).

The redox cofactor nicotinamide adenine dinucleotide (NAD+) plays a fundamental role in cellular energy production by carrying high energy electrons and driving oxidative phosphorylation (8). In addition to the *de novo* NAD+ biosynthesis pathway, NAD+ is biosynthesized from two additional pathways including the Preiss–Handler pathway, which makes NAD+ from dietary niacin, and the NAD+ salvage pathway which makes NAD+ from dietary or recycled niacinamide (Figures 1, 2). Despite the alternative pathways of NAD+ production, quinolinic acid accumulation is frequently accompanied by NAD+ reduction (9).

Over the past decade, a growing number of reports has described quinolinic acid accumulation during inflammation and has hypothesized that quinolinic acid may play a role in many disease processes. Inflammation triggers a complex cascade of cytokines which can activate the kynurenine pathway and cause quinolinic acid accumulation. In fact, IDO, the enzyme responsible for the first and rate-limiting step of this pathway, is induced by many inflammatory cytokines (10, 11). Interferon-γ (IFN-γ), specifically, is a powerful IDO-activating cytokine (12). Quinolinic acid seems to have a controversial and unclear role during inflammation. In some instances, quinolinic acid appears to have an anti-inflammatory role by reducing Th1-like cells and increasing the Th2-like cells, which limits adaptive immunity overactivation (13). However, quinolinic acid accumulation has also been considered a deleterious feature of inflammation, and its increased concentration can be responsible for cytotoxicity, particularly in neurologic diseases through numerous proposed mechanisms (2).

Quinolinic acid has been noted to accumulate in several organs, blood, cerebrospinal fluid (CSF), and urine during pathological conditions. This accumulation could be interpreted as either evidence of a high-flux state or as suppression of pathway enzymes, including QPRT activity, during inflammation and stress, leading to altered NAD+ biosynthesis (7, 14). Quinolinic acid accumulation has been associated with a spectrum of diseases including neurodegenerative conditions, psychiatric diseases, acute illness, kidney failure, and liver failure. The exact implication of quinolinic acid accumulation as well as its correlation with the disease severity is still uncertain. Urinary measurement of quinolinic acid is a convenient diagnostic test of this metabolite as it is a non-invasive and easily collected. Additionally, there is evidence that urinary quinolinic acid levels correspond with systemic levels (15). Several studies have assessed urinary quinolinic acid levels as markers of various pathologies. However, until now, a broad examination of urinary quinolinic acid excretion has not been undertaken. In this review, we categorize and summarize primary research that has reported quinolinic acid in human urine.

## Materials and methods

### Literature search

This study was guided by the Preferred Reported Items for Systematic Reviews and Meta-Analysis (PRISMA) statement issued in 2020. The search was restricted to English language journal articles with human subjects, published between December 1949 and January 2022.

The search of the literature was conducted using electronic databases PubMed, Cochrane, and Scopus. The following keywords were used to perform the search: ((Quinolinic Acid) OR (Quinolinate)) AND ((Urine) OR (Urinary) OR (Urine analysis)).

### Inclusion and exclusion criteria

Eligible studies were included if they were (i) peer reviewed primary scientific articles and (ii) reported quinolinic acid measurement in the urine of human subjects irrespective of study aim. Studies were excluded if they were (i) reviews or meta-analyses, (ii) articles written in languages other than English, (iii) assessments of quinolinic acid in body fluids other than urine, and (iv) studies performed exclusively on animals.

### Study selection and data extraction

Two investigators performed the literature search and selected qualified studies according to the inclusion and exclusion criteria. The study selection was performed in a two-step process, beginning with a title and abstract screening followed by full-text screening. The data extracted from each study included the general aim of the study, study method and design for measuring metabolites, and the results concerning quinolinic acid measurement in urine. Included studies were categorized in a system-based classification: endocrinology, gastroenterology, hematology, infectious diseases, nephrology, neurology, obstetrics and gynecology, oncology, psychiatry, rheumatology, and other conditions. A descriptive analysis of the results was presented.

## Results

The electronic database search yielded a total of 512 articles. Based on title and/or abstract, 90 articles were deemed potentially relevant, and after the full text viewing, 54 articles were included in the review (Figure 2).

Studies measuring urinary quinolinic acid in humans are shown in Table 1. Various methods were used to measure urinary quinolinic acid. However, most studies described chromatography-spectrometry techniques. Additionally, human urine exhibits significant variability in concentration and composition within an individual arising from various external and internal factors such as hydration, solute intake, and kidney function. To avoid false data interpretation, several normalization methods exist for urinary metabolomic studies (16). We specified for each study which normalization technique was used (if any) to insist on the validity of the results and to facilitate the interpretation of the data.

**TABLE 1:** Studies measuring urinary quinolinic acid in humans.

References	Disease or condition studied	N	Method for QA measurement in urine	Aim of the study	Results regarding quinolinic acid (QA) in urine	QA normalization
Studies involving specific disease states						
Endocrinology						
Oh et al. (29)	Metabolic syndrome	345	Liquid chromatography-tandem mass spectrometry	Describe a liquid chromatography-tandem mass spectrometry-based method for the simultaneous targeted analysis of tryptophan metabolites in urine. Validate this method in patients with metabolic syndrome and comparing them to healthy controls.	Urinary QA was higher in patients with metabolic syndrome compared to healthy controls.	Creatinine
Haam et al. (30)	Metabolic syndrome	529	High-performance liquid chromatography–mass spectrometry analyses	Investigate urinary organic acid metabolites in patients with metabolic syndrome.	Patients with metabolic syndrome showed higher urinary levels of QA.	Creatinine
Gastrointestinal						
Clària et al. (33)	Liver failure	50	Liquid chromatography, mass spectrometry	Investigate the kynurenine pathway in patients with acute on chronic liver failure (ACLF).	In ACLF patients, no significant difference was shown in urinary QA concentration between patients with and without kidney failure.	Fractional excretion
Genetic diseases						
Ney et al. (42)	Phenylketonuria	9	Metabolomics analysis by Metabolon Inc.	In patients with phenylketonuria, assess monoamine metabolites in patients with phenylketonuria consuming amino acid-based medical foods and compare them to those who consume glycosylated-peptides-based medical foods.	Urinary QA excretion was significantly higher with amino acid-based medical foods compared with glycosylated-peptides-based medical foods.	Urinary creatinine
Hematology						
Landfried et al. (24)	Graft vs. host disease	51	Liquid chromatography-tandem mass spectrometry.	Investigate the role of indoleamine 2,3-dioxygenase (IDO) in human allogeneic stem cell transplantation.	Urinary QA significantly increased with the severity of graft vs. host disease.	Urinary creatinine
Infectious disease						
Mason et al. (28)	Tuberculous meningitis	60	Gas chromatography–mass spectrometry	Identify metabolites that may diagnose Tuberculous meningitis in a non-invasive manner.	Urinary QA was significantly higher in patients with Tuberculous meningitis compared to control group.	Urinary creatinine
Nephrology						
Suhre et al. (43)	End stage renal disease	241	Combination of non-targeted liquid chromatography, mass spectrometry, and gas chromatography	Determine objective urine metabolites for predicting kidney allograft success and rejection.	Urinary QA increased significantly in patients whose kidney biopsies showed acute cellular rejection compared to those with no rejection.	No normalization was mentioned
Poyan Mehr et al. (6)	AKI	12	Liquid chromatography–mass spectrometry	Determine the efficacy of urinary quinolinic acid as an indicator of diminished *de novo* NAD+ biosynthesis	Urinary QA was higher in patients who developed AKI after cardiac surgery compared to those who did not develop AKI. In ICU patients, higher urinary QA correlated with increased illness severity and adverse outcomes including hospital mortality.	Tryptophan
Bajaj et al. (44)	AKI	435	Ultrahigh-performance liquid chromatography–tandem mass spectroscopy.	Investigate the role of metabolomics in the prediction of AKI and the need for dialysis in patients with hepatic cirrhosis.	Urinary QA was higher in patients who developed AKI compared to those who did not.	Tryptophan
Raines et al. (45)	AKI	46	Liquid chromatography–mass spectrometry.	Study the alterations of urine metabolomes in patients with COVID-19 associated AKI.	Patients with AKI had higher urinary QA: tryptophan ratio compared to patients with no AKI. Higher concentrations were found in patients requiring mechanical ventilation.	Tryptophan
Suhre et al. (46)	End stage renal disease-Kidney transplant	153	Liquid-phase mass-spectrometry analysis	Determine urinary metabolites that could identify kidney transplant rejection.	Urinary QA was significantly higher in patients with acute transplant rejection compared to those with no rejection.	Sample osmolality
Bignon et al. (7)	Acute to chronic kidney injury progression	41	Liquid chromatography–mass spectrometry	Determine the clinical relevance of non-invasive monitoring of QPRT activity in patients with kidney disease.	Urinary QA: tryptophan was higher at day 1 after surgery in patients who developed AKI and predicted progression to CKD in kidney transplant subjects.	Tryptophan
Neurology						
Heyes et al. (32)	Huntington’s disease	25	High pressure liquid chromatography	Measure QA excretion in patients with Huntington’s disease and compare them to age matched controls.	Urine QA was significantly lower in patients with Huntington’s disease compared to control groups. When QA was corrected as to the creatinine and BUN, no significant difference in urinary QA was found between groups.	Urinary creatinine and urea
Gevi et al. (26)	Autism spectrum disorder	60	Hydrophilic interaction chromatography, ultra-high-performance liquid chromatography, and mass spectrometry	Determine objective urinary metabolite markers of autism spectrum disorder (ASD) diagnosis.	Urinary QA was significantly higher in children with ASD compared to matched controls.	Urine specific gravity
Boczonadi et al. (20)	Mitochondrial oxodicarboxylate carrier deficiency	1	Ultrahigh-performance liquid chromatography—tandem mass spectrometry	Study mitochondrial oxodicarboxylate carrier deficiency in human and its effect on metabolites accumulation and neurological toxicity.	Mitochondrial oxodicarboxylate carrier deficiency led to QA accumulation in urine.	Porin
Mussap et al. (31)	Autism spectrum disorder	57	Gas chromatography-mass spectrometry	Determine if the severity of symptoms in autism is associated with specific metabolic alterations.	Urinary QA was significantly increased in children with autism compared to neurotypical children. Urinary QA was considerably higher in children with severely impaired behaviors compared to those with mild-to-moderate behaviors.	Urinary specific gravity
Amirdelfan et al. (47)	Chronic pain	487	Liquid chromatography–mass spectrometry	Validate mechanistic pain biomarkers	QA was significantly higher in patients suffering from chronic pain compared to healthy subjects.	Urinary creatinine
Harutyunyan et al. (48)	Autism spectrum disorder	24	Liquid chromatography, mass spectrometry, and spectrophotometry	Analyze different metabolic pathways, markers of immune system activation, and potential etiological factors related to ASD development	Urinary QA concentration was increased in ASD patients and positively correlated with T-helper lymphocyte level in blood.	Urinary creatinine
Obstetrics and gynecology						
Rose and Toseland (35)	Administration of deoxypyridoxine (vitamin B6 antagonist) in men and oral contraceptives in women	32	Gas-liquid chromatography	Determine the effect of deoxypyridoxine on urine QA excretion in healthy men and oral contraceptives in healthy women.	Urine QA was markedly increased after the administration of deoxypyridoxine in men and oral contraceptives in women.	No normalization was mentioned
Meloni et al. (17)	Premature rupture of membranes during pregnancy.	38	Gas chromatography–mass spectrometry	Determine the metabolites associated with premature rupture of membranes.	In women with premature rupture of membranes QA was higher during labor compared to out of labor.	No normalization was mentioned
Oncology						
Kim et al. (21)	Kidney cancer	62	Ultrahigh performance liquid chromatography-tandem mass spectrometry and gas chromatography-mass spectrometry	Assess for biomarkers of kidney cancer by comparing urine metabolomics from patients with kidney cancer to patients without cancer.	Urinary QA was higher in patients with kidney cancer.	Urine osmolality
Pasikanti et al. (34)	Bladder cancer	99	Two-dimensional gas chromatography time-of-flight mass spectrometry	Develop a non-invasive method to diagnose and follow-up bladder cancer.	The ratio of urinary tryptophan to QA was significantly lower in patients with bladder cancer compared to those without bladder cancer.	Urinary creatinine
Zimmer et al. (22)	Breast cancer	120	ELISA	Investigate the role of physical exercise in controlling the level of kynurenine pathway metabolites in breast cancer patients undergoing radiotherapy.	At baseline, urinary QA excretion was higher in women with breast cancer compared to healthy women. Moreover, QA decreased after exercise in both healthy women and women with breast cancer undergoing radiotherapy.	Urinary creatinine
Thüring et al. (23)	Prostate cancer	100	ELISA	Determine the prognostic value of indoleamine-2,3-dioxygenase gene expression in urine of patients with prostate cancer undergoing radical prostatectomy as first-line treatment.	QA: Tryptophan ratio positively correlated with Gleason score.	Tryptophan
Drago et al. (49)	Prostate cancer	50	Ultrahigh-performance liquid chromatography tandem mass spectrometry	Determine a metabolomic signature that may distinguish patients with clinically significant prostate cancer from those with benign prostatic hyperplasia.	QA was significantly higher in patients with prostate cancer compared to those with benign prostatic hyperplasia.	Probabilistic quotient normalization
Pediatrics						
Esturau-Escofet et al. (50)	Parenteral nutrition in preterm newborns	34	H nuclear magnetic resonance assay	Compare the effect of parenteral nutrition and enteral nutrition on the metabolomic profiles of preterm newborns.	Urinary QA did not significantly change between preterm newborns on parenteral nutrition compared to those on enteral nutrition.	Total area of the urine samples.
Psychiatry						
Banerjee and Agarwal (51)	Schizophrenia	20	High-performance liquid chromatography	Investigate differences in tryptophan metabolism between patients with schizophrenia and those without by assessing metabolomic responses to increased tryptophan intake.	Urinary QA was higher in patients with schizophrenia after the administration of tryptophan.	No normalization was mentioned
Zheng et al. (25)	Major depressive disorder	260	Gas chromatography-mass spectrometry	Determine objective markers for the diagnosis of major depressive disorder (MDD).	QA was significantly increased in the urine of patients with MDD compared to healthy patients.	Urinary creatinine
Chojnacki et al. (52)	Major depression disorder	90	Liquid chromatography with tandem mass spectrometry	Investigate tryptophan metabolism in elderly patients with mood disorders.	Elderly patients with moderate to severe depression showed higher urinary QA compared to a control group without mood disorders.	Urinary creatinine
Molina-Carballo et al. (27)	Attention deficit and/or hyperactivity disorder	179	Liquid-chromatography–tandem mass spectrometry	Investigate tryptophan and kynurenine metabolites in attention deficit and/or hyperactivity disorder (ADHD) children compared to healthy children and assess changes after treatment with methylphenidate (MPH) in blood and urine.	Urinary QA excretion was slightly lower in the control group compared to ADHD group. MPH treatment significantly decreased QA in urine of patients with ADHD (subgroup without depressive symptoms).	Urinary creatinine
Rheumatology						
Hankes et al. (53)	Scleroderma	6	Microbiological assay	Compare urinary metabolites of South African miners to American women with scleroderma after 2-g L-tryptophan load test and tracer doses of L-tryptophan-7a-14C, L-kynurenine-keto-14C and hydroxy-L-kynurenine-keto-14C.	The urinary excretion of QA was significantly elevated in the South African miners compared to American women with scleroderma.	No normalization was mentioned
Rider et al. (18)	Juvenile idiopathic inflammatory myopathies	124	Commercial ELISA, high performance liquid chromatography, or gas chromatography-mass spectrometry	Evaluate the utility of urinary neopterin and QA as markers of disease activity in juvenile idiopathic inflammatory myopathies.	Urine QA concentrations correlated with disease activity and increased with the severity of disease.	Urinary creatinine
Noakes (54)	Morphea	1	Gas chromatography-mass spectrometry	Study the effect of Tranilast on the urinary excretion of kynurenine metabolites in patients with morphea disease.	Administration of Tranilast decreased the urinary excretion of QA. The study suggested that Tranilast acts as a competitive inhibitor of indoleamine 2, 3-dioxygenase (IDO), tryptophan 2, 3-di-oxygenase (TDO), or both in the tryptophan pathway.	Urinary creatinine
Fernández-Ochoa et al. (19)	Sjögren’s syndrome	95	Mass spectrometry	Determine the metabolic pathways involved in Sjögren’s syndrome pathogenesis.	Urinary QA was higher in patients with Sjögren’s syndrome.	Mass spectrometry useful signal
Dietary and environmental manipulations						
Henderson et al. (1)	Tryptophan supplementation	4	Differential microbiological assay with lactobacillus arabinosus	Comparing quinolinic acid in urine of humans, guinea pigs, male calf, female lambs, male duroc jersey pigs before and after tryptophan ingestion.	QA rose markedly in urine during tryptophan administration.	No normalization was mentioned
Heeley et al. (55)	Pyridoxine supplementation	10	Spectrophotometry	Measure the excretion of QA with and without pyridoxine supplementation	The data could not show any significant difference in QA excretion with and without pyridoxine supplementation.	No normalization was mentioned
Brown et al. (56)	Vitamin B6 depletion	6	Microbiological assay	Investigate the effect of Vitamin B6 depletion on the conversion of tryptophan to nicotinic acid derivatives.	The urinary excretion of QA was higher in subjects with Vitamin B6 depleted diet. The level was restored to pre-depletion levels after the repletion in Vitamin B6.	No normalization was mentioned
Hankes et al. (57)	Vitamin B6 deficiency	11	Microbiological assay	Determine the effect of vitamin B6 deficiency and protein intake level on the excretion of QA in men.	The urinary excretion of QA increased during the deprivation period of Vitamin B6 and with increased intake of tryptophan.	No normalization was mentioned
Toseland (36)	Tryptophan supplementation	15	Gas-liquid chromatography	Measure QA in urine of men and women before and after tryptophan ingestion.	QA was markedly higher after tryptophan ingestion.	No normalization was mentioned
Crawford et al. (37)	Tryptophan supplementation	143	Ion-exchange chromatography	Assess the urinary metabolites of communities living in different regions of Uganda with different diets.	QA excretion rates in plantain eaters (plant rich in tryptophan) was considerably higher than non-plantain eaters.	No normalization was mentioned
Nakagawa et al. (58)	Vitamin B6 and valine supplementation	19	Microbiological assay	Assess the effect of excessive intake of leucine with and without addition of vitamin B6 and the effect of valine deficiency on the urinary excretion of different metabolites.	The excretion of QA was not affected by an excessive intake of leucine or a diet deficient in valine.	No normalization was mentioned
Krishnaswamy et al. (59)	Leucine and vitamin B6 supplementation	6	Microbiological assay	Assess the metabolic effect of dietary leucine and vitamin B6 supplementation on urinary quinolinic acid.	Dietary leucine supplementation increased quinolinic acid urinary excretion. Vitamin B6 counteracted the effect of leucine on quinolinic acid.	No normalization was mentioned
Yeh and Brown (60)	Vitamin B6 deficiency	6	Ion-exchange chromatography followed by specific colorimetric or fluorometric assays.	Assess the effect of vitamin B6 deficiency on the excretion of tryptophan metabolites in rats, guinea pigs, hamsters, and humans.	The urinary QA level was decreased in rats, unchanged in guinea pig and hamster, and increased in human with vitamin B6 deficiency?	No normalization was mentioned
Patterson et al. (61)	Tryptophan, leucine, and vitamin B6 supplementation	27	High pressure liquid chromatography	Assess the effect of tryptophan, leucine, and vitamin B6 intake on the excretion of tryptophan and niacin metabolites in men.	There was no significant effect of L-leucine or vitamin B6 supplementation on the urinary excretion of QA.	No normalization was mentioned
Hankes et al. (62)	Vitamin B6, riboflavin, thiamine, and vitamin C supplementation	6	Cation exchange chromatography column	Study the effect of dietary supplementation with vitamin B6, riboflavin, thiamin, and vitamin C on the conversion of tryptophan to niacin metabolites in patients with hepatoma.	Vitamin supplementation did not significantly affect QA excretion in urine.	No normalization was mentioned
Fukuwatari and Shibata (63)	Nicotinamide supplementation	6	High-performance liquid chromatography	Determine if *de novo* nicotinamide synthesis from tryptophan is influenced by nicotinamide intake in six women.	Urinary excretion of nicotinamide metabolites increased in a dose-dependent manner including QA with nicotinamide supplementation.	No normalization was mentioned
Hiratsuka et al. (64)	Tryptophan supplementation	17	High-performance liquid chromatographic	Determine the fate of dietary tryptophan in Japanese female adults.	QA excretion in urine was shown 18.9 ± 8.3 μmol/day.	No normalization was mentioned
Hiratsuka et al. (38)	Tryptophan supplementation	17	Liquid chromatography with fluorometric detection	Study the supplementation of tryptophan on the urinary excretion of multiple compounds including QA in healthy Japanese women.	Urinary excretion of QA increased in a dose dependent of tryptophan, but was unchanged in a time dependent manner.	No normalization was mentioned
Shibata et al. (65)	B-group vitamins on tryptophan metabolites	10	High-performance liquid chromatography	Investigate the effect of B-group vitamins (vitamin B1, B2, B3, B5, and B6) on tryptophan metabolites in Japanese adults.	The B-group vitamin (B1, B2, B3, B5, and B6) administration had no effect on tryptophan metabolites including QA excretion in urine.	Urinary creatinine
Poesen et al. (39)	Protein supplementation	29	Liquid chromatography–mass spectrometry	Investigate the effect of high protein intake on the mammalian metabolome (in mice and human).	QA was significantly higher in urine of subjects who were assigned to the high protein diet compared to the low protein diet.	Urinary creatinine
Nassan et al. (40)	Phthalates ingestion	30	Liquid chromatography and mass spectrometry	Study the association between phthalates and quinolinic acid in human.	High phthalate exposure increased urinary QA concentrations.	Urinary creatinine
Cao et al. (66)	High-intensity training	12	Gas chromatography-mass spectrometry	Explore the metabolic mechanism in teenage football players during exercise-induced fatigue.	QA significantly decreased in post-exercise group relative to the pre-exercise group	Internal standard normalization
Nassan et al. (67)	Dibutyl phthalate exposure	126	Liquid chromatography–mass spectrometry	Investigate the effect of low phthalate exposure on urinary QA.	Urinary QA increased with phthalate exposure.	Urinary creatinine
Oluwagbemigun et al. (68)	Temporal reproducibility	132	Ultra-high performance liquid chromatography coupled with electrospray ionization triple quadrupole mass spectrometry	Investigate tryptophan pathway in healthy adolescents at two points in time (1 year difference), retrieve strongly related metabolites and determine if they are temporarily reproducible.	Quinolinic acid was highly related to kynurenic acid and xanthurenic acid. No temporally reproducible association involved QA.	No normalization was mentioned

QA, quinolinic acid; ELISA, enzyme-linked immunosorbent assay; AKI, acute kidney injury.

Urinary quinolinic acid was measured in multiple pathological conditions. Compared to control groups, this metabolite was shown to be higher in urine of patients suffering from premature rupture of membranes during labor (17), juvenile idiopathic inflammatory myopathies (18), Sjögren’s Syndrome (19), mitochondrial oxodicarboxylate carrier deficiency (20), kidney cancer (21), breast cancer (22), prostate cancer (23), graft versus host disease (GVHD) (24), major depressive disorder (25), autism spectrum disorder (26), attention-deficit/hyperactivity disorder (ADHD) (27), tuberculous meningitis (28), kidney transplant rejection, acute kidney injury (AKI) (6), and metabolic syndrome including hyperlipidemia, obesity, hypertension, and diabetes (29, 30). In some cases, urinary quinolinic acid was even demonstrated to correlate with disease severity. In juvenile idiopathic inflammatory myopathies, urinary quinolinic acid was shown to increase with several aspects of disease activity including physician and parent global assessments of disease activity, manual muscle testing total score, Childhood Myositis Assessment Scale, and Childhood Health Assessment Questionnaire (18). In GVHD, urinary quinolinic acid was significantly higher in patients with GVHD grade 3 or 4 compared to those with grade 1 and 2 (24). Moreover, in a critically ill population, urinary quinolinic acid to tryptophan ratio was not only associated with higher risk of AKI development, but also increased probability of global adverse outcomes, including hospital mortality (6). In a study on patients with autism spectrum disorder comparing urine metabolomics of patients with severe autism spectrum disorder symptoms to those with mild-to-moderate symptoms, quinolinic acid was significantly higher in patients with severe symptoms (31). In patients with prostate cancer, quinolinic acid to tryptophan ratio level in urine significantly correlated with their Gleason score.

Conversely, Heyes et al. found no significant difference in urinary quinolinic acid in patients with Huntington’s disease compared to the control group (32). Patients suffering from acute on chronic liver failure showed no significant difference in their urinary quinolinic acid during acute kidney failure (33). And, one study investigating patients with bladder cancer showed a lower ratio of urinary tryptophan to quinolinic acid compared to the healthy control group (34).

Urinary quinolinic acid is increased in several non-disease settings as well. Quinolinic acid is reportedly higher in urine of patients who were administered deoxypyridoxine (a vitamin B6-antagonist), oral contraceptive (35), tryptophan (1, 36–38), high-protein diet (39), and those who were exposed to phthalate (an industrial chemical) (40; Table 1).

## Discussion

To our knowledge, this is the first systematic review evaluating urinary quinolinic acid in humans. There is abundant evidence that urinary quinolinic acid is increased in various disease states including cancer, infections, autoimmune diseases, metabolic syndrome, and psychiatric conditions, and in many instances quinolinic acid levels correlate with disease severity (41). While this review focused primarily on human urine, given its ease of measurement, this metabolite has also been investigated in blood, CSF, and tissue biopsies and has been implicated in the pathophysiology of different diseases in animals as well.

The included articles described a wide spectrum of settings in which quinolinic acid is increased with different methods and units of measurement. These variations limited our ability to compare findings. Another limitation to our systematic review is the difficulty of assessing bias in the included studies since the quinolinic acid data is not always primary outcome of the studies presented. We recognize that undetected biases may have been present in the articles described here. We also acknowledge that focusing on urinary-based quinolinic acid studies and excluding plasma and tissue-based quinolinic acid studies is an additional limitation to our results, but this was an indispensable filter for the purpose and feasibility of our study.

Overall, we were able to demonstrate build-up of quinolinic acid in urine in a wide spectrum of settings. Quinolinic acid increase during inflammation cannot be analyzed independently from its biochemical pathway, and the exact implication of its build-up is yet unrevealed. Acting as a double-edged sword, quinolinic acid is both an essential precursor for nicotinamide adenine dinucleotide (NAD+) biosynthesis and a potentially toxic metabolite at high concentrations. Quinolinic acid accumulation has been observed in numerous pathologic conditions. While most of the reported studies are observational in nature, they have highlighted that urinary measurement of quinolinic acid is an easy value to obtain that may reveal useful information about systemic disease processes. The true significance of its accumulation and its correlation with the severity of disease are still uncertain. Further studies are needed to investigate potential diagnostic, prognostic, and therapeutic implications of quinolinic acid.

## Data availability statement

The original contributions presented in this study are included in the article/supplementary material, further inquiries can be directed to the corresponding author.

## Author contributions

SP planned, designed, and supervised the project. AC and MS performed the literature search and collected the qualified studies according to the inclusion and exclusion criteria. All authors discussed the results, wrote the manuscript, and approved the submitted version.
